# The Endogenous Alterations of the Gut Microbiota and Feces Metabolites Alleviate Oxidative Damage in the Brain of LanCL1 Knockout Mice

**DOI:** 10.3389/fmicb.2020.557342

**Published:** 2020-10-07

**Authors:** Fangxing Zhang, Nana Qi, Yanyu Zeng, Mengying Bao, Yang Chen, Jinling Liao, Luyun Wei, Dehao Cao, Shengzhu Huang, Qianqian Luo, Yonghua Jiang, Zengnan Mo

**Affiliations:** ^1^Center for Genomic and Personalized Medicine, Guangxi Medical University, Nanning, China; ^2^Guangxi Key Laboratory of Genomic and Personalized Medicine, Nanning, China; ^3^Institute of Urology and Nephrology, First Affiliated Hospital of Guangxi Medical University, Nanning, China; ^4^Guangxi Collaborative Innovation Center for Genomic and Personalized Medicine, Nanning, China; ^5^Department of Urology, The First Affiliated Hospital of Guangxi Medical University, Nanning, China

**Keywords:** LanCL1, gut microbiota, feces metabolite, oxidative stress, gut-brain axis

## Abstract

Altered composition of the gut microbiota has been observed in many neurodegenerative diseases. LanCL1 has been proven to protect neurons and reduce oxidative stress. The present study was designed to investigate alterations of the gut microbiota in LanCL1 knockout mice and to study the interactions between gut bacteria and the brain. Wild-type and LanCL1 knockout mice on a normal chow diet were evaluated at 4 and 8–9 weeks of age. 16s rRNA sequence and untargeted metabolomics analyses were performed to investigate changes in the gut microbiota and feces metabolites. Real-time polymerase chain reaction analysis, AB-PAS staining, and a TUNEL assay were performed to detect alterations in the gut and brain of knockout mice. The serum cytokines of 9-week-old knockout mice, which were detected by a multiplex cytokine assay, were significantly increased. In the central nervous system, there was no increase of antioxidant defense genes even though there was only low activity of glutathione S-transferase in the brain of 8-week-old knockout mice. Interestingly, the gut tight junctions, zonula occludens-1 and occludin, also displayed a downregulated expression level in 8-week-old knockout mice. On the contrary, the production of mucus increased in 8-week-old knockout mice. Moreover, the compositions of the gut microbiota and feces metabolites markedly changed in 8-week-old knockout mice but not in 4-week-old mice. Linear discriminant analysis and *t*-tests identified *Akkermansia* as a specific abundant bacteria in knockout mice. Quite a few feces metabolites that have protective effects on the brain were reduced in 8-week-old knockout mice. However, N-acetylsphingosine was the most significant downregulated feces metabolite, which may cause the postponement of neuronal apoptosis. To further investigate the effect of the gut microbiota, antibiotics treatment was given to both types of mice from 5 to 11 weeks of age. After treatment, a significant increase of oxidative damage in the brain of knockout mice was observed, which may have been alleviated by the gut microbiota before. In conclusion, alterations of the gut microbiota and feces metabolites alleviated oxidative damage to the brain of LanCL1 knockout mice, revealing that an endogenous feedback loop mechanism of the microbiota-gut-brain axis maintains systemic homeostasis.

## Introduction

The gut microbiota, which contains 500–1000 species of commensal bacteria, is a huge system in the body and plays an important role in the maintenance of host systemic homeostasis, immune function, nutrient absorbance, and many other activities (Hooper and Gordon, 2001). In recent years, fecal metabolites have been found to play a crucial role in regulating the host’s health and systemic homeostasis (Koh et al., 2016), with significant effect on the nervous system. Numerous lines of evidence have determined that the gut microbiota is an essential regulator of the gut-brain axis and that it influences the function of the brain through nervous, immune, and endocrine pathways (Fülling et al., 2019).

Lanthionine synthetase components C (LanC) is involved in the synthesis of antimicrobial peptides. LanCL1, as a peripheral membrane protein that is notably expressed in the brain and testis, was first isolated from human erythrocyte membranes, and is regarded as a member of the eukaryotic LanC-like protein family (Mayer et al., 1998, 2001). Recent research has identified that LanCL1 is a glutathione binding protein, taking part in antioxidant activities and protecting neurons, which may have a role in neurodegenerative diseases (Chung et al., 2007). Moreover, the expression of LanCL1 is developmentally regulated and is induced by neuronal activity. Genetic deletion of LanCL1 causes enhanced oxidative stress and apoptotic neurodegeneration in the brain of mice (Huang et al., 2014).

An alteration of the gut microbial composition has been observed in many neurodegenerative diseases and mental disorders, such as Alzheimer’s disease and schizophrenia (Li et al., 2019; Wang et al., 2019; Xu et al., 2019). It was previously unknown whether changes of the gut microbiota and fecal metabolites occur in LanCL1 knockout (KO) mice, which experience oxidative damage to their brain. In the present study, we observed the systematic and local alterations of the LanCL1 KO mice. To track any changes in the guts of LanCL1 KO mice, 16S rRNA sequencing and liquid chromatography-tandem mass spectrometry (LC-MS/MS) were used to detect the compositions of the gut microbiota and metabolites and to identify the specific bacteria and metabolites. Furthermore, antibiotics treatment was also performed to delete any difference in the gut microbiome between wildtype (WT) and KO mice and to investigate any causal relationship between differential bacteria and oxidative damage to the brain. Finally, we revealed the presence of a feedback loop in the microbiota-gut-brain axis, which plays a key role in maintaining systemic homeostasis.

## Materials and Methods

### Animals

Animals were maintained and bred in a specific pathogen-free environment at 27°C, in a regular atmosphere, with a 12 h light/dark cycle, and with standard chow and water provided *ad libitum* at Guangxi Medical University Laboratory Animal Center. LanCL1 knockout mice were purchased from Shanghai Biomodel Organism Science and Technology Development Company Limited. Exon 4 of LanCL1 was excised using CRISPR/Cas9 technology based on non-homologous end joining (NHEJ). The deletion of the LanCL1 exon 4 led to a frame shift in the coding sequence, which caused complete knockout of the gene product. Founder mice were bred with wild-type C57BL/6J mice to establish a germline-transmitted mutant mouse line. Then, the mice were intercrossed to generate heterozygous offspring (LanCL1+/−), homozygous offspring (KO; LanCL1−/−), and littermate controls (WT; LanCL1+/+), which were identified by PCR genotyping (Supplementary Figures 1A,B). LanCL1 knockout mice were confirmed by Western blotting (Supplementary Figure 1C). All experimental mice were maintained and studied in parallel to minimize any environmental effects. Body weights were determined at 4, 8, and 11 weeks. Fecal samples were collected at 4 and 8 weeks, placed in sterile plastic tubes, and rapidly snap-frozen in liquid nitrogen, then stored at −80°C until used. Animals were sacrificed at 4, 8–9, and 11 weeks of age. Blood samples were collected via orbital puncture, and tissues (cecum and brain) were immediately collected and frozen in liquid nitrogen and stored at −80°C, or fixed with a 4% paraformaldehyde solution. The study was approved by The Medical Ethics Committee of The First Affiliated Hospital of Guangxi Medical University.

### PCR Genotype Assays

Genomic DNA of mice was extracted from mouse tail tips by the routine phenol-chloroform method and subjected to PCR amplification followed by agarose gel examination. All mice were genotyped using the following PCR primers: P1: 5-GGAAATGCTTTAGGCAGACAG-3, P2: 5-GCAACTCC ACCTGCTGACA-3, and P3: 5-CAGCGATGCCTGGAATGT-3. The primers were paired as P1 + P2 and P1 + P3 to identify the genotype, as shown in the schematic diagram in Supplementary Figure 1A. Wild-type mice were identified by the P1 + P2 band of 1371 bp and the P1 + P3 band of 690 bp, heterozygous mice were identified by two P1 + P2 bands of 500 bp and 1371 bp and a P1 + P3 band of 690 bp, and knockout mice were identified by only a P1 + P2 band of 500 bp and no P1 + P3 band (Supplementary Figure 1B). The following PCR program was used for PCR genotyping: 94°C for 5 min, 35 cycles of 94°C for 30 s, 62°C for 30 s and 72°C for 1.5 min, then 72°C for 5 min and 12°C for holding.

### Western Blot

Tissues were lysed using a radio-immunoprecipitation assay (RIPA) lysis buffer. The collected supernatant was quantified using a bicinchoninic acid (BCA) protein assay (Thermo Fisher Scientific, United States) and boiled with loading buffer for 5 min to denature the proteins. The proteins were loaded onto sodium dodecylsulfate-polyacrylamide (10% SDS-PAGE) gels, separated by electrophoresis, and transferred onto a polyvinylidene difluoride (PVDF) membrane. The PVDF membrane was blocked with 2% non-fat dry milk for 2 h at room temperature, soaked in a TBS-Tween-20 (TBST) solution containing primary antibodies (LanCL1, 1:1000, Proteintech; GAPDH, 1:1000, CST) overnight at 4°C, rinsed with TBS to wash away unbound primary antibodies, and exposed to a TBST solution containing peroxidase-labeled secondary antibodies (1:5,000; no. ab6721; Abcam) for 1 h at room temperature. After washing away unbound secondary antibodies, the chemiluminescent method was used to detect protein expression.

### Open-Field Test

The open-field test was performed to evaluate the locomotion and anxiety-related behaviors of WT and KO mice at 8 weeks (Takeda et al., 1998). The open field apparatus was a 40 cm × 40 cm square with 40 cm-high walls. The illumination intensity was 80 l× during the test. The software (SMART; Panlab, SL, Barcelona, Spain) divided the open field into 16 equal-size squares and detected the body shape and motion trail of mice. Four squares in the center were defined as the central area, and the other 12 squares were defined as the peripheral area. Mice were transported to the testing room 1 h before the test. Each mouse was placed in the central area and tested for 6 min. The distance traveled and time spent in the central and peripheral areas were recorded by the software (SMART; Panlab, SL, Barcelona, Spain) during the test. Between each test, the apparatus was cleaned with 75% ethanol.

### Antibiotic Treatment

WT and KO mice were divided into four groups for treatment (WT, WT+ antibiotics, KO, and KO+ antibiotics). Antibiotic treatment was provided as previously described (Sampson et al., 2016). In short, animals were treated with vancomycin (0.5 g/L; Shanghai Yuanye Bio-Technology), ampicillin (1 g/L; Shanghai Yuanye Bio-Technology), gentamycin (100 mg/L; Shanghai Yuanye Bio-Technology), erythromycin (10 mg/L; Shanghai Yuanye Bio-Technology), and neomycin (0.5 g/L; Shanghai Yuanye Bio-Technology), which were administered via drinking water for 6 weeks, from 5 to 11 weeks of age.

### Total RNA Preparation, cDNA Synthesis, and Quantitative Reverse Transcription PCR (qRT-PCR)

Total RNA was isolated with an E.Z.N.A.TM Total RNA Kit (Omega). For each tissue type, before and after antibiotic treatment, experiments were performed on three different samples. Following isolation, reverse transcription to cDNA was performed using a PrimeScript^TM^ RT reagent kit (TaKaRa). qRT-PCR analysis was performed on a Roche LightCycler^®^96 qRT-PCR system with TB Green^TM^Premix Ex Taq^TM^II (TaKaRa), and GAPDH was used as an endogenous control. The qRT-PCR primers used are listed in Supplementary Table 1.

### Detection and Analysis of Inflammation Factors in Mice

Inflammation factors in mice were detected and analyzed with the LEGENDplex^TM^ Mouse Inflammation Panel, which is a multiplex assay that uses fluorescence-encoded beads suitable for analyzing various flow cytometers. This panel allows for simultaneous quantification of 13 mouse cytokines, including IL-1α, IL-1β, IL-6, IL-10, IL-12p70, IL-17A, IL-23, IL-27, MCP-1, IFN-β, IFN-γ, TNF-α, and GM-CSF. The panel was validated by detecting the expected changes in biological samples. The results were analyzed by the LEGENDplex^TM^ Data Analysis Software.

### Immunohistochemical Analysis

Immunohistochemical (IHC) analysis was performed on 4% paraformaldehyde fixed and paraffin embedded (FFPE) tissue samples with specific antibodies. The samples were cut to a thickness of 3 μm and dried at 60°C for 30 min. The specificity of the antibody (anti-LanCL1, 1:200, Proteintech) was tested using the cecum of mice. The sections were deparaffinized and rehydrated in a dewaxing solution. Antigen retrieval was conducted with a citrate buffer solution (pH 6.5) at 95°C for 20 min. Endogenous peroxidase was blocked with a hydrogen peroxide solution for 4 min. The diluted primary antibody was incubated with the tissue for 12 h at 4°C. Next, the secondary antibody, a rabbit anti-mouse antibody, was applied a rabbit anti-mouse antibody (1:200, Solarbio). The slides were visualized using colorimetric detection with diaminobenzidine (DAB). Samples were counter-stained with hematoxylin. Finally, all slides were scanned and photographed in a scanner.

### Alcian Blue and Periodic Acid-Schiff Staining

Alcian blue and periodic acid-Schiff (AB-PAS) staining were performed with the panel (Solarbio, G1285). The prepared sections were treated with Alcian blue dye for 5 min after deparaffinization and rehydration, washed with running water for 2 min, and stained with periodic acid for 5 min. The slices were treated for 30 min with Schiff’s stain under protection from light. After washing with running water, the sections were subjected to hematoxylin staining, followed by dehydration through xylene. Images were obtained under a computer-supported imaging system connected to a light microscope.

### TUNEL Assay

TdT-mediated dUTP nick end labeling (TUNEL) was used to detect cell apoptosis in the tissues. The Colorimetric TUNEL Apoptosis Assay kit (Beyotime) was used according to the manufacturer’s protocol. Apoptotic cells were visualized using colorimetric detection with diaminobenzidine (DAB). Images were obtained under a computer-supported imaging system connected to a light microscope.

### 16S rRNA Gene Sequencing Analysis

The gut microbiome samples were sent to the NOVOGENE Company Limited, China (Beijing, China) for DNA extraction and sequencing of the 16S rRNA gene according to the manufacturer’s instructions. Total genomic DNA from the samples was extracted using the CTAB/SDS method. DNA concentration and purity were monitored on 1% agarose gels. DNA was diluted to 1 ng/μL using sterile water. 16S rRNA genes from distinct regions (16S V3–V4) were amplified using specific primers with a barcode. All PCR reactions were carried out in 30 μL with 15 μL of Phusion^®^ High-Fidelity PCR Master Mix (New England Biolabs), 0.2 μM of the forward and reverse primers, and approximately 10 ng template DNA. Thermal cycling consisted of an initial denaturation at 98°C for 1 min, followed by 30 cycles of denaturation at 98°C for 10 s, annealing at 50°C for 30 s, and elongation at 72°C for 30 s, and finally, 72°C for 5 min. Then, the mixture of PCR products was purified with a GeneJETTM Gel Extraction Kit (Thermo Scientific). Sequencing libraries were generated using the Ion Plus Fragment Library Kit 48 rxns (Thermo Scientific) following the manufacturer’s recommendations. The library quality was assessed using a Qubit@ 2.0 Fluorometer (Thermo Scientific). Finally, the library was sequenced on the Ion S5TM XL platform, and 400/600-bp single-end reads were generated.

After data clean-up, sequences with 97% similarity were assigned to the same OTUs. Representative sequences for each OTU were screened for further annotation. Alpha diversity was applied to analyze the complexity of the species diversity for a sample through 5 indices, including Observed-species, Chao1, Shannon, Simpson, and ACE. All the indices in our samples were calculated with QIIME (Version1.7.0). Non-Metric Multi-Dimensional Scaling (NMDS) and Unweighted Pair-group Method with Arithmetic Means (UPGMA) Clustering were calculated by QIIME software (Version 1.7.0) and displayed by the vegan and ggplot2 package in R software (Version 2.15.3). UPGMA was performed as a type of hierarchical clustering method to interpret the distance matrix using the average linkage. Linear discriminant analysis effect size (LEfSe) was used to investigate bacterial members that drive differences between groups. LEfSe software was used to visualize the results.

### Untargeted Metabolomics Analysis

The gut microbiome samples were sent to NOVOGENE Company Limited, China (Beijing, China), for metabolite extraction and liquid chromatography-tandem mass spectrometry (LC-MS/MS) analyses according to the manufacturer’s instructions. Feces samples were individually ground in liquid nitrogen, and the homogenate was resuspended in prechilled 80% methanol and 0.1% formic acid by vortexing. The samples were incubated on ice for 5 min and were then centrifuged at 15000 rpm, 4°C for 5 min. The supernatant was diluted to a final concentration containing 60% methanol by LC-MS grade water. The samples were subsequently transferred to a fresh Eppendorf tube through a 0.22 μm filter and were then centrifuged at 15000 *g*, 4°C for 10 min. Finally, the filtrate was injected into the LC-MS/MS system for analysis. LC-MS/MS analyses were performed using a Vanquish UHPLC system (Thermo Fisher) coupled with an Orbitrap Q Exactive HF-X mass spectrometer (Thermo Fisher).

After the identification, quantitative results were obtained, and quality control was assessed to ensure the accuracy and reliability of the data. Partial least squares discriminant analysis (PLS-DA) was performed by SIMCA-P software 11.5 (Umetrics AB, Umea, Sweden) to analyze the metabolite data to reveal differences in the feces metabolic composition between the WT and KO groups. The Variable Importance in the Projection (VIP) value of the first principal component of the PLS-DA model can identify differentially expressed metabolites. A volcano plot was displayed by the ggplot2 package in R software (Version 2.15.3) based on the threshold value: VIP > 1, the difference multiple FC > 2.0 or FC < 0.5, and *P*-value < 0.05. Kyoto Encyclopedia of Genes and Genomes (KEGG) enrichment analysis based on the hypergeometric algorithm was performed to identify the most specific KEGG pathway, displayed by the ggplot2 package in R software (Version 2.15.3). The heatmap of the Spearman correlation between the top 10 bacteria and the top 20 metabolites (based on *P*-value) was displayed by the corrplot package in R software (Version 2.15.3).

### Statistical Analysis

Data are presented as the mean ± SEM. Significance between two groups was determined by Student’s *t*-test. Datasets that involved more than two groups were analyzed by one-way analysis of variance (ANOVA), followed by Tukey’s multiple comparisons test. Data with a non-normal distribution were analyzed with the Mann-Whitney *U*-test in SPSS 22.0 (SPSS Inc., Chicago, IL, United States). ImageJ v1.52 (W. Rasband, Open Source) was used to analyze 2–3 representative images of each mouse. The total pixel area of the colonic crypts or brain sections in every image was measured. The positive staining area was isolated by color contrast manipulation, and the pixel area was measured. The proportions of the positive area in the colonic crypt area or brain section were calculated for all images of KO and WT mice. Differences were noted as significant *P < 0.05, **P < 0.01, ***P < 0.001, ****P < 0.0001. Figures were generated by GraphPad Prism 8.0.2 (GraphPad Software, Inc.). All experiments were repeated at least three times.

## Results

### Loss of LanCL1 Causes Significant Differences in Body Weight and the Serum Levels of Cytokines but Not in Oxidative Stress of the Brain

The body weight of wildtype (WT) and LanCL1 knockout (KO) mice was measured at 4, 8, and 11 weeks of age. Compared with WT mice, LanCL1 KO mice showed a statistically significant decrease of body weight at 11 weeks, which was not found at 4 and 8 weeks (Supplementary Figure 2A). As organs with high level expression of LanCL1 (Mayer et al., 1998), the brain weight of KO mice was significantly higher than WT mice at 4 weeks, and the testis weight of the KO mice was reduced significantly at 11 weeks. There were no significant differences at other time points.

LanCL1 is regarded as being similar to glutathione S-transferase (GSTs) and plays a role in GSH-mediated antioxidant defense (Huang et al., 2014). Loss of LanCL1 reduces GST activity in the brain of KO mice and aggravates oxidative damage in the brain (Chung et al., 2007; Huang et al., 2014). Here, we found that the expression of major GST enzymes (GSTP1, GSTM1, and GSTA4) in the brain showed a decreasing trend from 4 to 8 weeks (Supplementary Figure 2B). However, the antioxidant defense genes, including PGC-1α, PGC-1β, SOD-1, and SOD-2, were not upregulated in the brain of LanCL1 KO mice at either 4 or 8 weeks of age, contrary to the prediction based on the low activity of GSTs. The movement and anxiety-related behaviors between the two types of mice showed no differences either (Supplementary Figure 2C).

The typical tight junction proteins of the gut, zonula occludens-1 (ZO-1) and occludin, also displayed a decreasing trend of expression from 4 to 8 weeks in KO mice (Supplementary Figure 2D). Moreover, we assessed systemic inflammation by detecting the serum cytokines of WT and KO mice at 4 and 9 weeks. Compared with WT mice, the serum levels of IFN-γ, IFN-β, TNF-α, MCP-1, IL-17A, IL-1α, IL-6, and IL-12p70 were significantly increased in 9-week-old KO mice, while there were no significant differences between WT and KO mice at 4 weeks of age (Supplementary Figure 2E).

Collectively, all these findings further confirmed that development-dependent dysfunction was occurring in LanCL1 KO mice.

### Loss of LanCL1 Alters the Composition of the Gut Microbiota

As a high level of systemic inflammation and downregulated expression of tight junction proteins were found in LanCL1 KO mice, we wondered whether the composition of the gut microbiome was altered in KO mice since it plays an important role in regulating systemic homeostasis.

**FIGURE 1:**
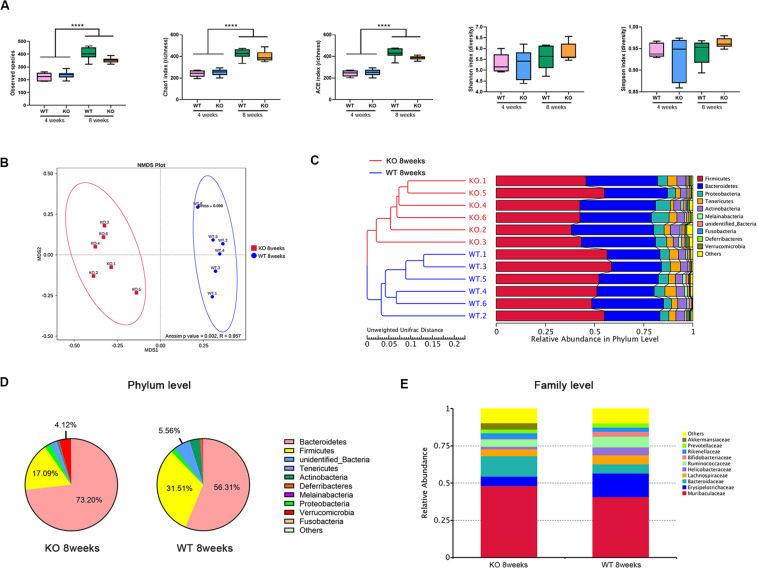
Loss of LanCL1 induces an altered composition of the gut microbiota at 8 weeks of age. **(A)** The box-plot of alpha diversity indices including the observed species, Chao1, ACE, Shannon, and Simpson indexes. **(B)** Non-metric multi-dimensional scaling (NMDS) based on the Bray-Curtis distance presents the relationship between the microbial profiles of the two groups at 8 weeks of age. NMDS has good reliability when stress <0.2. The *R*-value of the Anosim analysis is in the interval (–1, 1). *R*-value > 0 indicates that the differences between groups were significant. *R*-value < 0 indicates that the difference within groups is greater than the difference between groups. **(C)** The unweighted pair-group method with arithmetic mean (UPGMA) based on the Unweighted UniFrac distance was used to determine the degree of similarity between the gut microbiota of WT and KO mice at 8 weeks of age. **(D)** Fan diagram showing the relative abundance of bacterial 16S rRNA genes classified at the phylum level. **(E)** The bacterial compositions of different communities at the family level. Taxa with abundances <1% are included in others. A significant difference (*P* < 0.05) was determined by one-way ANOVA followed by Tukey’s multiple comparisons test for **A**. For **A** through **E**, *n* = 6/group.

In total, 1,816,725 high-quality sequences from 24 gut microbiome samples were obtained, with an average of 70697 sequences per sample. Those sequences were delineated into 408 and 700 operational taxonomic units (OTUs) at 97% similarity for the 4 and 8 weeks groups, respectively. Alpha-diversity was determined by calculating the following indexes: for the observed species, the Chao1 and ACE indexes are relative to the microbial community richness, while the Shannon and Simpson indexes mainly reflect their evenness and homogeneity. It has been reported that the alpha-diversity of the gut microbiota increases with age, likely resulting from increased exposure to environmental microbiota over time (Flemer et al., 2017; Hoffman et al., 2017). Here, we observed that for both types of mice, the microbial community at 4 weeks of age was less species-rich than that at 8 weeks, which means the species-richness increases markedly over time. However, there were no differences observed in the richness or evenness indexes between WT and KO mice at the same ages (Figure 1A).

To evaluate the beta diversity between WT and KO mice, which reflects the degree of comparability in different microbial communities, non-metric multi-dimensional scaling (NMDS) and Anosim analyses were performed on the basis of the Bray-Curtis distance. NMDS analysis displayed a significant separation between WT and KO mice at 8 weeks of age (Figure 1B). Moreover, the unweighted pair-group method with arithmetic mean (UPGMA) based on the Unweighted UniFrac distance was used to assemble a clustering tree. The tree showed that WT and KO mice at 8 weeks were divided clearly into two groups (Figure 1C).

At the phylum level, the top five main phyla that comprised the gut microbiota of WT mice were: *Bacteroidetes, Firmicutes, Proteobacteria, Actinobacteria*, and *Deferribacteres* (Figure 1D). Compared to WT mice of the same age, the abundance of *Bacteroidetes* and *Verrucomicrobia* were markedly increased in 8-week-old KO mice, with a decrease of abundance of *Firmicutes.* At the family level, the relative abundance of *Akkermansiaceae* significantly increased in 8-week-old KO mice (*P* = 0.019) (Figure 1E). However, there were no such clear classifications or distinct changes between 4-week-old KO and WT mice (Supplementary Figure 3).

Taken together, all of these results revealed that the loss of LanCL1 led to a strong alteration of the structure of the gut microbial with increasing age, especially after 8 weeks, which paralleled the development-dependent dysfunction of in LanCL1 KO mice.

### Specific Bacteria in the Gut Microbiota of LanCL1 KO Mice May Affect the Production of Mucus in Their Goblet Cells

**FIGURE 2:**
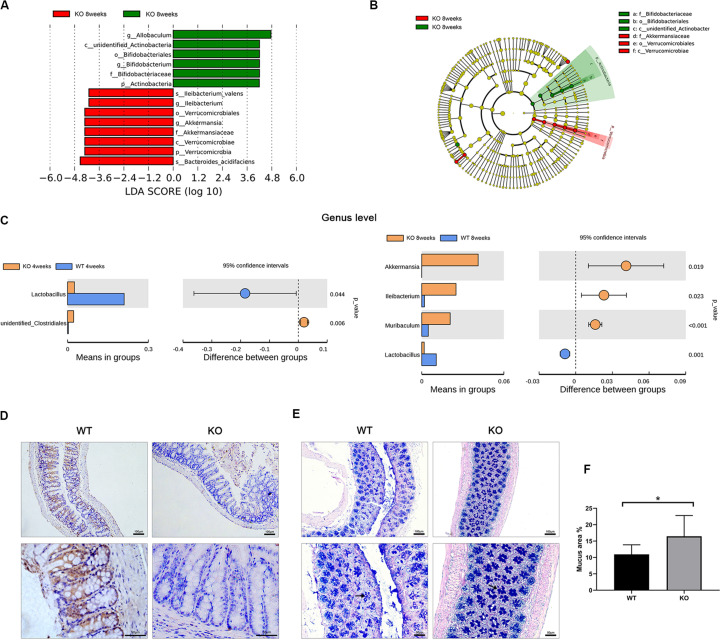
Identified specific bacteria and alterations of the gut structure in LanCL1 KO mice. **(A,B)** Linear discriminant analysis effect size (LEfSe) comparison of microbiota in fecal samples of mice at 8 weeks. LDA scores and cladogram generated from the LEfSe analysis, showing the bacterial taxa that were differentially abundant in LanCL1 KO mice at 8 weeks of age. **(C)** *T*-tests were performed at the genus level to identify specific bacteria between the two types of mice at 4 and 8 weeks. **(D)** A representative image of LanCL1 immunostaining in the cecum of 8-week-old mice. Scale bar: upper 100 μm, lower 50 μm. **(E,F)** A representative image of AB-PAS staining in the cecum of 8-week-old WT and KO mice and the relative proportion of the area of mucus. Scale bar: upper 100 μm, lower 50 μm. Significant differences (*P* < 0.05) were determined by LEfSe analysis by both the Kruskal Wallis test (among classes) and the Wilcoxon test (between subclasses) for **(A,B)**. The threshold logarithmic LDA score was 4.0. A significant difference (*P* < 0.05) was determined by unpaired two-tailed Student’s *t*-test for **(C,F)**. For A through **(C)**, *n* = 6/group. For **(D)** through **(F)**, *n* = 3–4/group.

To identify the specific bacteria that characterized the gut microbiome of 8-week-old LanCL1 KO mice, the linear discriminant analysis effect size (LEfSe) was calculated. A linear discriminant analysis (LDA) score >4.0 was the screening condition for the analysis, and the cladogram showed the specific flora as microbiological markers between WT and KO mice at multiple phylogenetic levels. Interestingly, most of the specific flora clustered into two distinct branches of the phylogenetic tree: *Akkermansiaceae* (phylum *Verrucomicrabia*) was markedly plentiful in KO mice, while *Bifidobacteriaceae* (phylum *Actinobacteria*) was a significant microbiological marker in WT mice (Figures 2A,B).

*T*-tests between the two groups were also performed to find the specific bacteria at the genus level, and there were some interesting findings (Figure 2C). Compared to WT mice, *Lactobacillus* was markedly reduced in KO mice in both age groups. This result means that the loss of LanCL1 may lead to a specific reduction in the abundance of *Lactobacillus*. Moreover, the phylogenetic branch of *Akkermansia* was significantly plentiful in 8-week-old KO mice, similar to the results of the LEfSe analysis. Enrichment of *Ileibacterium* and *Muribaculum* at the genus level was also detected in 8-week-old KO mice.

Since the gut microbiome in 8-week-old KO mice was altered, histological analysis was used to ascertain whether the LanCL1 protein was expressed in the gut and whether the loss of LanCL1 affected the structure of the gut. The expression of LanCL1 was measured by an IHC method. As shown in Figure 2D, there were no significant structural changes observed in the cecum of KO mice, and the LanCL1 protein was detected around goblet cells in WT mice but was not detected in KO mice. Interestingly, AB-PAS staining showed that the production of mucus in goblet cells was markedly increased in KO mice (*P* < 0.05; Figures 2E,F), while no major change was observed in the relative number of goblet cells.

It has been reported that *Akkermansia* can promote the production of mucins (Derrien et al., 2017). Here, we hypothesized that the loss of LanCL1 may alter the composition of the gut microbiota and further affect the production of mucins in goblet cells.

### Alteration of Metabolites in Feces From LanCL1 KO Mice at 8 Weeks of Age

The gut microbiota could influence the host via microbial metabolites, which is known as the host-microbe metabolic axis (Pi et al., 2017). We wanted to further assess the metabolic alterations of feces from 8-week-old KO mice. Fecal metabolites are strongly regulated by the gut microbiota.

**FIGURE 3:**
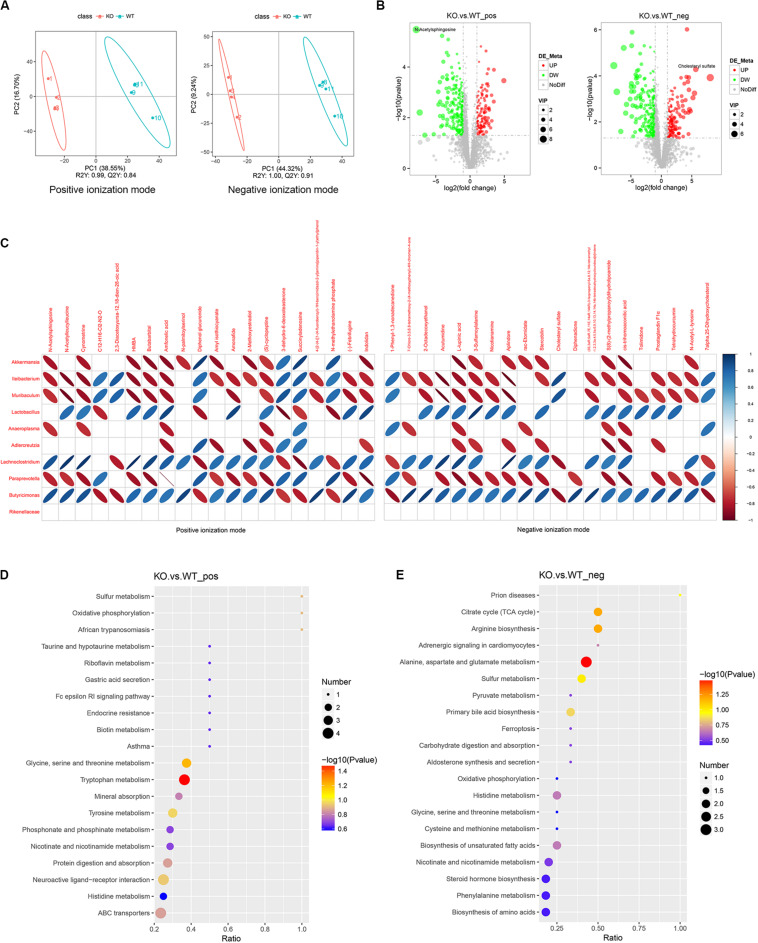
Alteration of feces metabolites is observed in LanCL1 KO mice. **(A)** Partial least squares discriminant analysis (PLS-DA) score plots for discriminating the fecal metabolome between 8-week-old WT and KO mice in the positive and negative ion modes. **(B)** Volcano plot displaying the composition of the differentially expressed metabolites in the positive and negative ion modes between 8-week-old WT and KO mice. The threshold value: VIP > 1, the difference multiple FC > 2.0, or FC < 0.5, *P*-value < 0.05. **(C)** Heatmap analysis of the Spearman correlation between the top 10 specific bacteria and the top 20 specific metabolites in the positive and negative ion modes. Only the significant correlations (*P* ≤ 0.05) are shown as colored marks in the chart. Blue represents a positive correlation, and red indicates a negative correlation. For A through C, *n* = 4/group. **(D,E)** KEGG enrichment analysis of differentially expressed metabolites in the positive and negative ionization modes. For A through E, *n* = 4/group.

Partial least squares discriminant analysis (PLS-DA) showed a significant separation between WT and KO mice in both the positive and negative ionization modes (Figure 3A). The R2Y (predictive power) and Q2Y (explanatory power) as the evaluation parameters of the PLS-DA model were closer to 1, which means that the model is reliable.

Widespread changes of the metabolites were detected in KO mice. A total of 3,430 positive ion-mode metabolites were identified, including 130 significantly over-expressed metabolites and 241 significantly downregulated metabolites. A total of 2,875 negative ion-mode metabolites were identified, including 153 significantly over-expressed metabolites and 232 significantly downregulated metabolites (Figure 3B). As shown in the volcano plot, N-acetylsphingosine was the most differentially expressed metabolite among the decreased metabolites, while cholesteryl sulfate was specifically differentially expressed metabolite among the increased metabolites. The other significant differentially expressed metabolites in the volcano figure were selected and are listed in Supplementary Tables 2, 3. As shown in the tables, most of the differentially expressed metabolites were decreased in KO mice. It is worth noting that, in both the positive and negative ion modes, taurine was identified as a specifically decreased metabolite. Short-chain fatty acids (SCFAs), as key gut metabolites, have various effects on host health and disease (Koh et al., 2016; Nicolas and Chang, 2019). The SCFAs detected by LC-MS/MS are listed in Supplementary Table 4, which shows an increased production of propionic acid in KO mice.

To assess the correlation between the gut microbiota and fecal metabolites, the top 20 differentially expressed metabolites from the positive and negative ion-modes and the top 10 differential bacteria were collected to perform Spearman’s correlation analysis. As the heatmap of the correlation shows, *Akkermansia*, *Ileibacterium*, and *Muribaculum*, as the markedly plentiful microbiota in 8-week-old KO mice, were negatively correlated with most of the differentially expressed metabolites, while *Lactobacillus*, as the microbiological marker of WT mice, was positively correlated with most of differentially expressed metabolites (Figure 3C).

KEGG pathway analysis revealed that differentially expressed metabolites in both ion-modes were mainly enriched in pathways of the citrate cycle, arginine biosynthesis, alanine, aspartate and glutamate metabolism, glycine, serine and threonine metabolism, and tryptophan metabolism (Figure 3D–E). These significant pathways were mostly clustered in the annotations of global and overview maps and amino acid metabolism (Supplementary Figure 4).

### Alteration of the Gut Microbiome May Alleviate Oxidative Damage in the Brain of LanCL1 KO Mice

**FIGURE 4:**
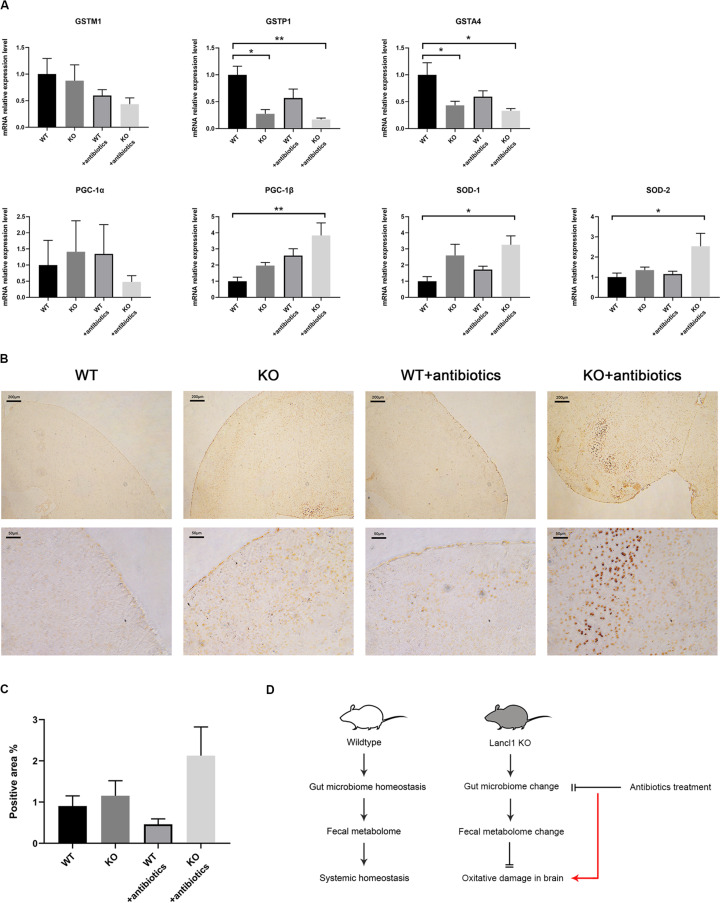
Effects of antibiotics treatment on the central nervous system. **(A)** mRNA expression of glutathione S-transferase M1 (GSTM1), glutathione S-transferase P1 (GSTP1), glutathione S-transferase A4 (GSTA4), proliferator-activated receptor γ coactivator-1α (PGC-1α), proliferator-activated receptor γ coactivator-1α (PGC-1β), superoxide dismutase-1 (SOD-1), and superoxide dismutase-2 (SOD-2) in the brain of the two types of mice after antibiotics treatment was quantified by qRT-PCR and normalized against GAPDH (*n* = 3–4/group). **(B,C)** Representative image of TUNEL staining in the brain of 11-week-old WT and KO mice that received the antibiotics treatment and the relative proportion of the positively stained area (*n* = 3–4/group). Scale bar: upper 200 μm, lower 50 μm. **(D)** Schematic representation of the model proposed in this work. A significant difference (*P* < 0.05) was determined by one-way ANOVA followed by Tukey’s multiple comparisons test for **A** or by the unpaired two-tailed Student’s *t*-test for **C**.

As alterations of the gut microbiome and fecal metabolome were observed in 8-week-old KO mice, we tried to associate these alterations with the phenomenon found in the brain of LanCL1 KO mice, that antioxidant defense genes were not upregulated in response to increased oxidative stress. Did the change of the gut microbiome downregulate oxidative stress in the brain? To test this hypothesis, we treated KO and WT mice with gentamycin, ampicillin, erythromycin, vancomycin, and neomycin, which were administered via drinking water from 5 to 11 weeks of age, to remove the differences in the gut microbiome (Sun et al., 2019). After 6 weeks of treatment with the cocktail of antibiotics, the expression levels of relevant genes in the brain were detected again. A low activity of GST enzymes was still observed in KO mice, and the expression of GSTM1 and GSTA4 was significantly downregulated in all KO mice, which was not influenced by the antibiotics (Figure 4A). Compared to WT mice, interestingly, the expression of antioxidant defense genes (PGC-1β, SOD-1, and SOD-2) were significantly upregulated in the KO mice that received the antibiotics treatment. To further confirm this result, as a histological detection method of cell apoptotic death, TUNEL staining of the brain was performed. Consistently, the brains of treated LanCL1 KO mice displayed increased TUNEL positive areas in the cortex and cerebellum (Figures 4B,C). These findings supported our hypothesis that the alteration of the gut microbiome in LanCL1 KO mice changed the fecal metabolome and further alleviated oxidative damage in the brain. The antibiotics treatment that deleted the regulatory ability of the gut microbiome led to the aggravation of oxidative damage in the brain of LanCL1 KO mice (Figure 4D).

## Discussion

A large amount of research has shown that the gut microbiome and feces metabolites can strongly influence host health and the central nervous system (Nicholson et al., 2012). In this study, the results clearly confirmed that gut microbial communities in 8-week-old mice changed in response to the loss of LanCL1, and the specific bacteria *Akkermansia* and *Lactobacillus* were identified. Moreover, the fecal metabolome demonstrated significant changes that may further regulate the host. More importantly, a regulative ability of the gut microbiome to alleviate oxidative stress in the brain of LanCL1 KO mice was observed, as proven by the treatment with antibiotics. These findings support the important role of the microbiota-gut-brain axis in regulating systemic homeostasis, notably in the central nervous system.

In our study, the weight of the testis was significantly decreased in 11-week-old KO mice, whose body weight was also decreased. Moreover, we observed that the brain weight in KO mice seemed to be increasing faster than that in WT mice at young ages. No differences in brain weight were observed at older ages, and the behavior and motility of KO mice were normal. It has been reported that LanCL1 is expressed highly in the testis and brain. Loss of LanCL1 may increase oxidative stress in the entire body, especially the testis, and brain. The antioxidant system plays a key role in the development of the testis, since spermatogenesis and Leydig cell steroidogenesis are sensitive to the effects of oxidative stress (Aitken and Roman, 2009). In LanCL1 KO mice, increased oxidative damage may lead to testicular dysfunction and further influence the development of the entire body through subsequent changes in the endocrine and immune system. In addition, there is much evidence suggesting that the loss of LanCL1 promotes oxidative damage in the nervous system (Huang et al., 2014; Tan et al., 2019), but due to other homeostasis effects, its loss does not seem to result in dramatic changes to the nervous system.

The gut barrier consists of the gut epithelium, tight junction proteins, the mucus layer, antimicrobial peptides, and so on (Hussain et al., 2019). Damage to the gut barrier can lead to systemic irritation and inflammation (Camilleri, 2019). In LanCL1 KO mice, expression of the tight junction occludin shows significant downregulation at 8 weeks, which may suggest a defect in gut barrier function. The reduction of gut tight junction proteins was caused by a high-fat diet, which also induced increased levels of IL-1β, TNF-α, IL-6, and IFN-γ in the serum (Hussain et al., 2019). *Dictyophora indusiata* polysaccharide can promote the recovery of the function of the intestinal barrier and reduce the levels of IL-1β, TNF-α, and IL-6 in serum after antibiotic-driven dysbiosis (Kanwal et al., 2018). In our study, increased pro-inflammation cytokines in serum, including IFN-γ, TNF-α, IL-1α, and IL-6, were observed in KO mice at 9 weeks of age, which may be associated with a defect in the gut barrier or increasing systemic oxidative stress.

LanCL1 is a member of the eukaryotic LanC-like protein family that plays a role in the synthesis of antimicrobial peptides (Mayer et al., 2001). Antimicrobial peptides (AMPs) are regarded as components of the innate immune response, and they act to kill invasive bacteria and prevent colonization by pathogens (Zaiou, 2007). In the mouse colon, goblet cells secrete the most AMPs to the mucus layer to separate bacteria from the surface of epithelial cells and to shape the bacterial communities in mucus (Jarret et al., 2020). As we found in 8–9-week-old KO mice, loss of LanCL1 in the entire body causes dysfunction in the synthesis of AMPs, which may lead to alterations of the gut microbial structure.

Surprisingly, the production of mucus was significantly increased in LanCL1 KO mice compared to WT mice. Mucus, as the first physical defense of the colon, plays an important role in preventing antigens, toxins, and bacteria from entering the body (Camilleri, 2019). AMPs and other immune regulators are released into the mucus gel (Johansson and Hansson, 2016). The release of colonic mucus is mediated by the enteric nervous system, enteroendocrine cells, and resident immune cells (Plaisancié et al., 1998). More interestingly, the gut microbiota can also shape the colon mucus barrier (Jakobsson et al., 2015). The LanCL1 protein was detected around goblet cells, which suggested that LanCL1 is associated with the synthesis of AMPs in the colon. Since the reduction of AMPs could not explain the high production of mucus, these findings imply that the gut microbiota plays a key role in increasing the production of mucus in KO mice.

As presented in this study, the most obvious change in the gut microbiota was observed at the phylum level, where the *Firmicutes*/*Bacteroidetes* ratio markedly decreased in 8-week-old KO mice. It has been reported that the *Firmicutes*/*Bacteroidetes* ratio is positively correlated with obesity (Ley et al., 2006; Turnbaugh et al., 2006), which can also explain the decreased weight of 11-week-old KO mice.

Moreover, *Lactobacillus* significantly decreased in KO mice from 4 to 8 weeks, and meanwhile, *Bifidobacterium* was markedly reduced in 8-week-old KO mice. *Bifidobacterium* and *Lactobacillus* are representative probiotic bacteria and common inhabitants of the human intestine, both of which have been proven to beneficially affect human health through different mechanisms (Turroni et al., 2014). *Lactobacillus* and *Bifidobacterium* strains have anti-proliferative, proapoptotic, and anti-oxidant properties, which lead to various anti-cancer actions (Nowak et al., 2019). A decrease in the abundance of those two types of bacteria in LanCL1 KO mice further indicates that neurodegenerative disease and systematic dysfunction could result in the loss of probiotics in the gut.

However, it was very interesting that there was a significant increase of *Akkermansia* in 8-week-old KO mice. *Akkermansia* is another important probiotic that has shown beneficial effects on many systematic dysfunctions, including diabetes, liver disease, colitis, aging, and even amyotrophic lateral sclerosis (ALS) (Hansen et al., 2012; Kang et al., 2013; Grander et al., 2018; Blacher et al., 2019; Bárcena et al., 2019). Interestingly, *Akkermansia* is also increased in the gut microbiota of patients with Parkinson’s disease (Nishiwaki et al., 2020). *Akkermansia* actively communicates with the host immune system, with potential anti-inflammatory responses, promotes barrier integrity, and potentially modulates the resident gut microbiota (Derrien et al., 2017). *Akkermansia*, which regulate glucose and lipid metabolism, are negatively correlated with many metabolic syndromes, such as diabetes and obesity (Greer et al., 2016; Xu et al., 2020). Host-derived mucins are the carbon and nitrogen sources of *Akkermansia*, which it decomposes into acetic, propionic, and butyric acid (Derrien et al., 2004; Xu et al., 2020). Those short-chain fatty acids (SCFAs) or other metabolites produced by *Akkermansia* can influence glucose and lipid homeostasis and even systemic homeostasis (Chambers et al., 2018). It is worth noting that the abundance of *Akkermansia* is positively correlated with the thickness of mucus in the cecum (Van den Abbeele et al., 2011). *Akkermansia* can promote the production of mucus and improve gut barrier function (Reunanen et al., 2015). As the study by Ijssennagger et al. shows, a decrease of *Akkermansia* abundance resulting from antibiotic treatment causes a reduction in the expression of the major mucin of colonic mucus and reduced mucolysis (Ijssennagger et al., 2015). *Akkermansia* has a positive effect on the amelioration of metabolic responses and the restoration of the gut barrier through regulation of the thickness of the mucus layer (Ottman et al., 2017). Based on our findings, we believe that *Akkermansia* plays a crucial role not only in restoring the gut barrier but also in alleviating the oxidative damage in the brain of KO mice.

It is not surprising that a large alteration of feces metabolites occurred in 8-week-old KO mice as an effect of the change in their gut microbial composition. The gut microbiome is a huge and complex system that regulates homeostasis mostly through the metabolites it produces (Li et al., 2014). As displayed in Supplementary Tables 2, 3, most of the specific metabolites were reduced. Among the most significantly differentially expressed metabolites, quite a few metabolites are associated with the central nervous system and mental system.

Taurine was a specific reduced metabolite of KO mice in both modes, and it has a protective effect on the nervous system. Niu et al. showed that taurine can protect injured brain cells from inflammation, apoptosis, and oxidative stress in *in vitro* experiments (Niu et al., 2018). Many researchers have reported the potential ameliorating effects of taurine against different neurological disorders, such as depression, anxiety, stroke, spinal cord injuries, and neurodegenerative diseases (Jakaria et al., 2019).

Caffeic acid phenethyl ester, another specific reduced metabolite in KO mice, has neuroprotective properties. Injections of caffeic acid phenethyl ester decrease apoptosis of brain cells in pentylenetetrazole-induced status epilepticus rats (Yiş et al., 2013).

In addition, as components of psychotropic drugs, minaprine and acamprosate were also significantly reduced in KO mice. Minaprine is an atypical antidepressant drug, and acamprosate is used to treat alcohol dependence. Both of these drugs exert neuroprotective effects on the central nervous system (Kodama et al., 1992; Choi et al., 2019).

Most of the altered metabolites clustered in pathways of amino acid metabolism in our study, and most of the final products of amino acid metabolism are short-chain fatty acids (SCFAs) (Neis et al., 2015). SCFAs, as key metabolites, can improve host health and regulate the function of various systems, such as the gut, nervous, and endocrine systems, by reducing inflammation, regulating immune reactions, protecting the gut barrier, and even regulating the gut-brain axis (Nicolas and Chang, 2019). In our study, propionic acid, as a representative SCFA, was overexpressed in the feces of KO mice. It has been widely reported that the enteric bacterial metabolite propionic acid can induce neuroinflammation and oxidative stress in the CNS (Shultz et al., 2008; Khalil et al., 2015). Furthermore, propionic acid is often used as an inducer of an animal model of autism, so the neurotoxic effects of propionic acid are believed to play a central role in the etiology of autism (MacFabe et al., 2007; El-Ansary et al., 2012).

The most eye-catching metabolite was N-acetylsphingosine, which was the most significantly reduced metabolite in KO mice. N-acetylsphingosine is a type of sphingosine and is also referred to as C2-Ceramide, a lipid second messenger. The level of ceramide increases after proapoptotic stimuli and during aging (Jana et al., 2009). As many studies have reported, C2-ceramide can lead to neuronal death and even cerebellar cell death by inducing apoptosis and autophagy (Minano et al., 2008; Zhang et al., 2012; Jaramillo-Gómez et al., 2015), and it has been considered to be a possible cause of many neurodegenerative disorders. Specific bacteria in KO mice were negatively correlated with N-acetylsphingosine in our study, and these results imply that specific gut microbiota negatively affected the production of N-acetylsphingosine. Meanwhile, the increase of cholesteryl sulfate was also marked. Cholesteryl sulfate may affect the biochemical activity of sphingosine (Downing et al., 1993), suggesting an interaction between cholesteryl sulfate and N-Acetylsphingosine in KO mice.

The broad decrease of feces metabolites that have protective effects on the brain further proved that the loss of LanCl1 is associated with dysfunction of the central nervous system. However, the decrease of N-Acetylsphingosine in KO mice, as the most significant alteration induced by the gut microbiota, may result in the postponement of neuronal apoptosis and protection of the brain. In short, the gut microbiota plays a critical role in the regulation of the nervous system.

The interactions between the gut microbiota and nervous system have been thoroughly researched. The gut microbiota and feces metabolites regulate intestinal immunity (Spencer et al., 2019) and further affect the enteric nervous system (ENS) and even the central nervous system (CNS). The important role of the microbiota-gut-brain axis in regulating neural inflammation and dysfunction is not a new notion. There are various ways for the gut bacteria and the brain to communicate (Huh and Veiga-Fernandes, 2019). The ENS, which senses gut bacteria, has a physical connection to the CNS. The metabolites or small molecules produced by the gut bacteria can diffuse to the brain and directly affect the CNS. Moreover, the peripheral immune cells affected by gut bacteria can send signals to the CNS.

An increasing number of studies have identified the regulative ability of the microbiota-gut-brain axis. Probiotic supplementation can reduce neuroinflammation in aging and improve brain function (Boehme et al., 2019). The gut microbiota of alpha-synuclein-overexpressing mice (a model of Parkinson’s Disease) promotes motor dysfunction and neuroinflammation, while antibiotic treatment ameliorates the pathophysiology in animals, revealing that postnatal signaling between the gut and the brain modulates this disease (Sampson et al., 2016). In our study, we observed that the microbiota-gut-brain axis regulated neuroinflammation via postnatal signaling without probiotic supplementation, and the antibiotic treatment that deleted the regulative ability of the gut microbiota aggravated oxidative damage in the brain. This endogenous performance may reveal a feedback-loop mechanism in the microbiota-gut-brain axis, which plays a key role in maintaining systemic homeostasis.

LanCL1 protects neurons against anti-oxidative stress. LanCL1 has been proven to protect neurons in amyotrophic lateral sclerosis (ALS) mice (Tan et al., 2019), while the gut microbiota can also ameliorate the symptoms of ALS mice (Blacher et al., 2019). Furthermore, LanCL1, as a potential prostate cancer (PCa) susceptibility gene, can also protect PCa cells from oxidative stress and promotes cell proliferation (Wang et al., 2018). A high-fat diet can alter the composition of the gut microbiome and promote prostate carcinogenesis in a transgenic mouse prostate model (Liu et al., 2019). It is noteworthy that chronic prostatitis, as an inducer of prostate cancer (Roberts et al., 2004), frequently induces mental disorders, such as anxiety and depression, and alterations of brain function and structure (Anderson et al., 2008; Farmer et al., 2011). The roles that LanCL1 and the gut microbiota play in chronic prostatitis are worthy of further exploration.

There are some limitations to our study. First, the gut microbiota and feces metabolite studies were based on small numbers of each type of mouse (*N* = 4–6/group). Larger samples may be more beneficial to achieve more precise results. Second, the gut microbiota at the species level were not fully investigated due to the sequence length. Third, although distinct alterations of the microbiota and metabolites and associations between the gut bacteria and brain were observed, the potential mechanisms still need to be further elucidated.

## Conclusion

In the present study, we observed the location of the expression of LanCL1 in the gut and investigated the effects of LanCL1 on the gut microbiome and feces metabolites. Loss of LanCL1 can induce some changes in the integrity of the gut barrier as well as alterations in the gut microbial community and metabolites. Interestingly, these alterations in the gut micro-environment have some positive effects on the central nervous system and even on systemic homeostasis. Our findings provide new evidence on the interactions between the gut microflora and the host, suggesting that *Akkermansia* is a probiotic with beneficial effects on the reduction of oxidative stress and protection of the CNS. Furthermore, we confirmed that the feces metabolites, which are closely related to the gut microbiota, have important functions in regulating the CNS. All of these results provide a new perspective and therapeutic targets for the treatment of neurodegenerative diseases. More importantly, a potential feedback loop mechanism of the microbiota-gut-brain axis to maintain systemic homeostasis was observed, which needs to be further explored.

## Data Availability Statement

The original contributions presented in the study are publicly available. This data can be found here: https://www.ncbi.nlm.nih.gov/bioproject/630864, BioProject ID is PRJNA630864.

## Ethics Statement

The animal study was reviewed and approved by the Medical Ethics Committee of First Affiliated Hospital of Guangxi Medical University.

## Author Contributions

FZ, YC, YJ, and ZM contributed to the conception and design of the study. YC, LW, DC, SH, QL, and JL performed the generation of LanCL1−/− mice. FZ, NQ, YZ, MB, and JL performed the experimentation. FZ and NQ performed mainly the write-up. All authors actively participated in research work and contributed to the manuscript and approved the submitted version.

## Conflict of Interest

The authors declare that the research was conducted in the absence of any commercial or financial relationships that could be construed as a potential conflict of interest.

## Supplementary Material

Supplementary Figure 1 **(A)** Illustration of the design of the LanCL1 targeting construct and the theory of identifying the genotype. **(B)** Result of PCR genotyping from agarose gel electrophoresis. **(C)** Western blots showing the absence of the LanCL1 protein in the knockout mice.

Supplementary Figure 2 **(A)** Body weight (g), brain weight (mg), and testis weight (mg) measured at 4, 8, and 11 weeks of age for both types of mice (*n* = 6/group). **(B)** mRNA expression of glutathione S-transferase M1 (GSTM1), glutathione S-transferase P1 (GSTP1), glutathione S-transferase A4 (GSTA4), proliferator-activated receptor γ coactivator-1α (PGC-1α), proliferator-activated receptor γ coactivator-1α (PGC-1β), superoxide dismutase-1 (SOD-1) and superoxide dismutase-2 (SOD-2) in the brain of 4 and 8-weeks-old mice was quantified by qRT-PCR and normalized against GAPDH (*n* = 3–4/group). **(C)** The distance travelled and time spent in different areas of the open field test (*n* = 5–6/group). **(D)** mRNA expression of Zonula Occludens-1 (ZO-1) and Occludin in the cecum of 4- and 8-weeks-old mice were quantified by qRT-PCR and normalized against GAPDH (*n* = 4/group). **(E)** The serum level of interferon-γ (IFN-γ), interferon-β (IFN-β), tumour necrosis factor-α (TNF-α), monocyte chemoattractant protein-1 (MCP-1), interleukin-17A (IL-17A), interleukin-1α (IL-1α), interleukin-6 (IL-6), and interleukin-12p70 (IL-12p70) was detected by a multiplex cytokine assay (*n* = 3/group). Data are presented as the mean ± SEM. A significant difference (*P* < 0.05) was determined by unpaired two-tailed Student’s *t*-test for **(A,B,D)**, and **(E)** or by the Mann-Whitney test for **(C)** (only performed between the same type or same age of mice).

Supplementary Figure 3 **(A)** Non-metric multi-dimensional scaling (NMDS) presenting the relationship between the microbial profiles of the two groups at 4 weeks of age. **(B)** Unweighted pair-group method with arithmetic mean (UPGMA) based on the Unweighted UniFrac distance was used to determine the degree of similarity between the gut microbiota of WT and KO mice at 4 weeks of age **(C)**. Fan diagram of the relative abundance of bacterial 16S rRNA genes classified at the phylum level **(D)**. Bacterial compositions of different communities at the family level. Taxa with abundances <1% are included in Others. A significant difference (*P* < 0.05) was determined by one-way ANOVA followed by Tukey’s multiple comparisons test for **(A)**. For A through **(G)**, *n* = 6/group. **(E,F)** Linear discriminant analysis effect size (LEfSe) comparison of microbiota from faecal samples of mice at 4 weeks. LDA scores and cladogram generated from the LEfSe analysis showing the bacterial taxa that were differentially abundant in LanCL1 KO mice at 4 weeks of age.

Supplementary Figure 4 Altered pathway annotations induced by differential metabolites in the positive and negative ionization modes.

Supplementary Table 1 Gene primers used in the study.

Supplementary Table 2 The differential feces metabolites of KO mice in positive ion mode.

Supplementary Table 3 The differential feces metabolites of KO mice in negative ion mode.

Supplementary Table 4 The short chain fatty acids detected in feces metabolites of mice.
