# Supplementary figures and images for: The Endogenous Alterations of the Gut Microbiota and Feces Metabolites Alleviate Oxidative Damage in the Brain of LanCL1 Knockout Mice
Source: Front Microbiol. 2020 Oct 7;11:557342. doi: 10.3389/fmicb.2020.557342 (PMC7575697; doi:10.3389/fmicb.2020.557342)

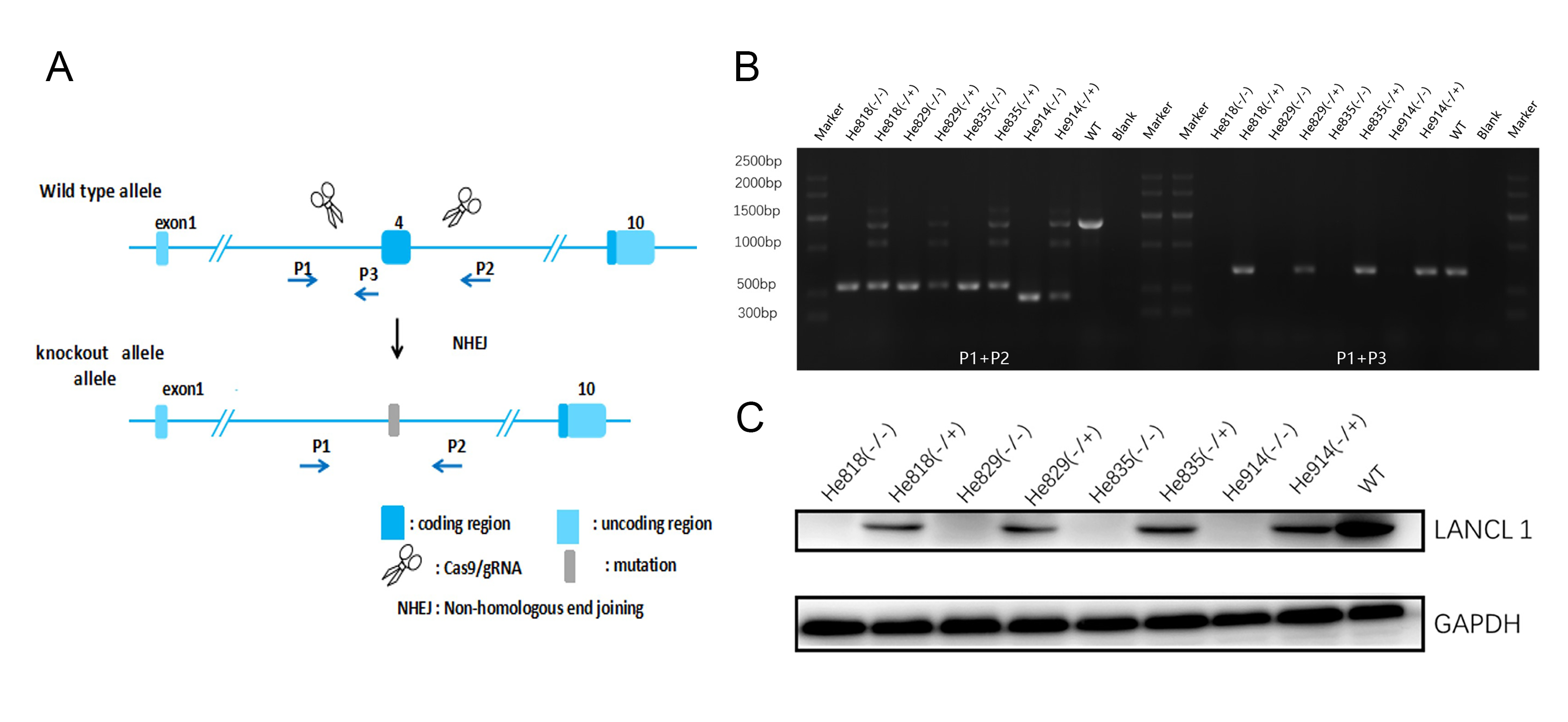

Supplement: Supplementary Figure 1 — (A) Illustration of the design of the LanCL1 targeting construct and the theory of identifying the genotype. (B) Result of PCR genotyping from agarose gel electrophoresis. (C) Western blots showing the absence of the LanCL1 protein in the knockout mice. [file Image_1.TIF]

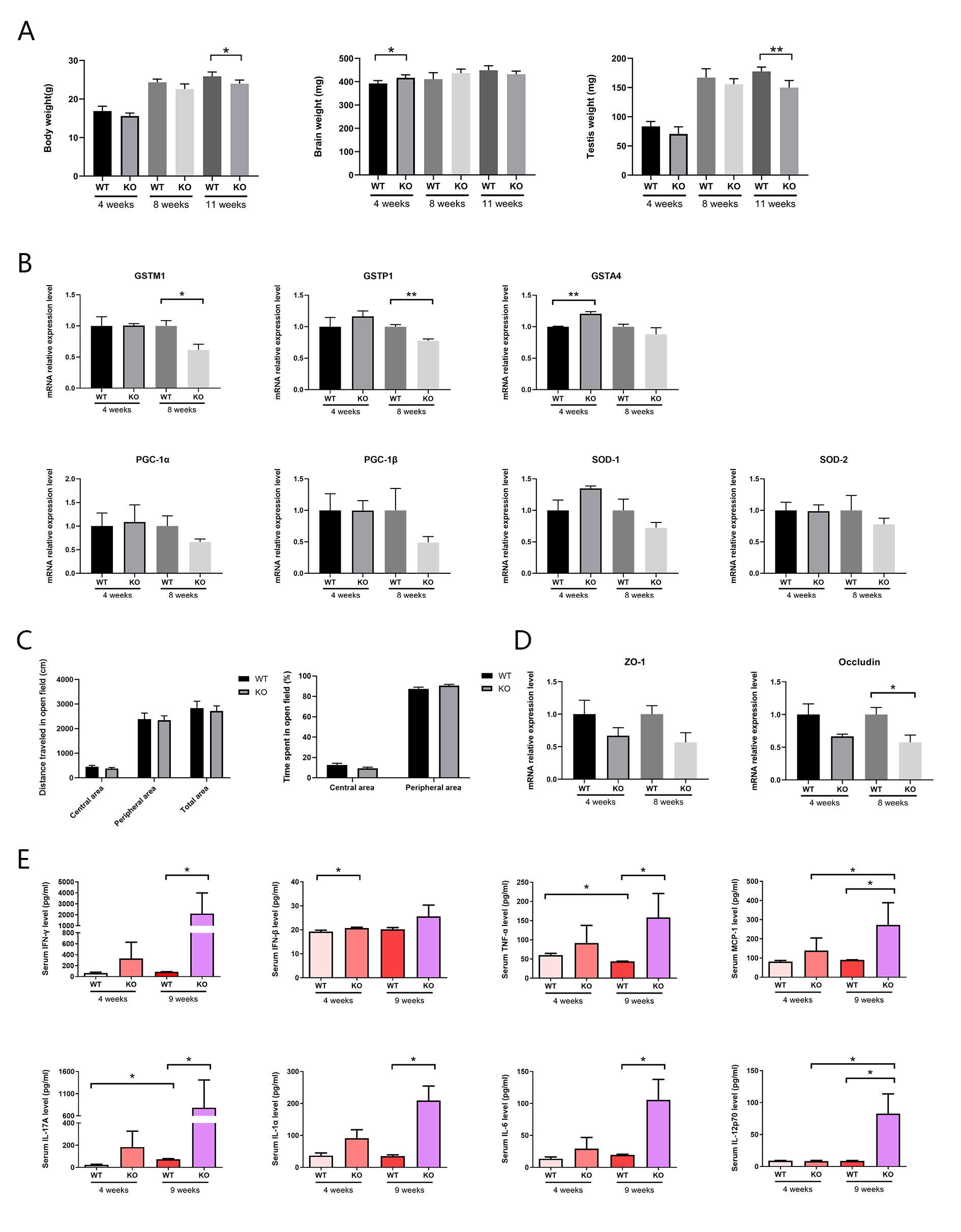

Supplement: Supplementary Figure 2 — (A) Body weight (g), brain weight (mg), and testis weight (mg) measured at 4, 8, and 11 weeks of age for both types of mice (n = 6/group). (B) mRNA expression of glutathione S-transferase M1 (GSTM1), glutathione S-transferase P1 (GSTP1), glutathione S-transferase A4 (GSTA4), proliferator-activated receptor γ coactivator-1α (PGC-1α), proliferator-activated receptor γ coactivator-1α (PGC-1β), superoxide dismutase-1 (SOD-1) and superoxide dismutase-2 (SOD-2) in the brain of 4 and 8-weeks-old mice was quantified by qRT-PCR and normalized against GAPDH (n = 3–4/group). (C) The distance travelled and time spent in different areas of the open field test (n = 5–6/group). (D) mRNA expression of Zonula Occludens-1 (ZO-1) and Occludin in the cecum of 4- and 8-weeks-old mice were quantified by qRT-PCR and normalized against GAPDH (n = 4/group). (E) The serum level of interferon-γ (IFN-γ), interferon-β (IFN-β), tumour necrosis factor-α (TNF-α), monocyte chemoattractant protein-1 (MCP-1), interleukin-17A (IL-17A), interleukin-1α (IL-1α), interleukin-6 (IL-6), and interleukin-12p70 (IL-12p70) was detected by a multiplex cytokine assay (n = 3/group). Data are presented as the mean ± SEM. A significant difference (P < 0.05) was determined by unpaired two-tailed Student’s t-test for (A,B,D), and (E) or by the Mann-Whitney test for (C) (only performed between the same type or same age of mice). [file Image_2.TIF]

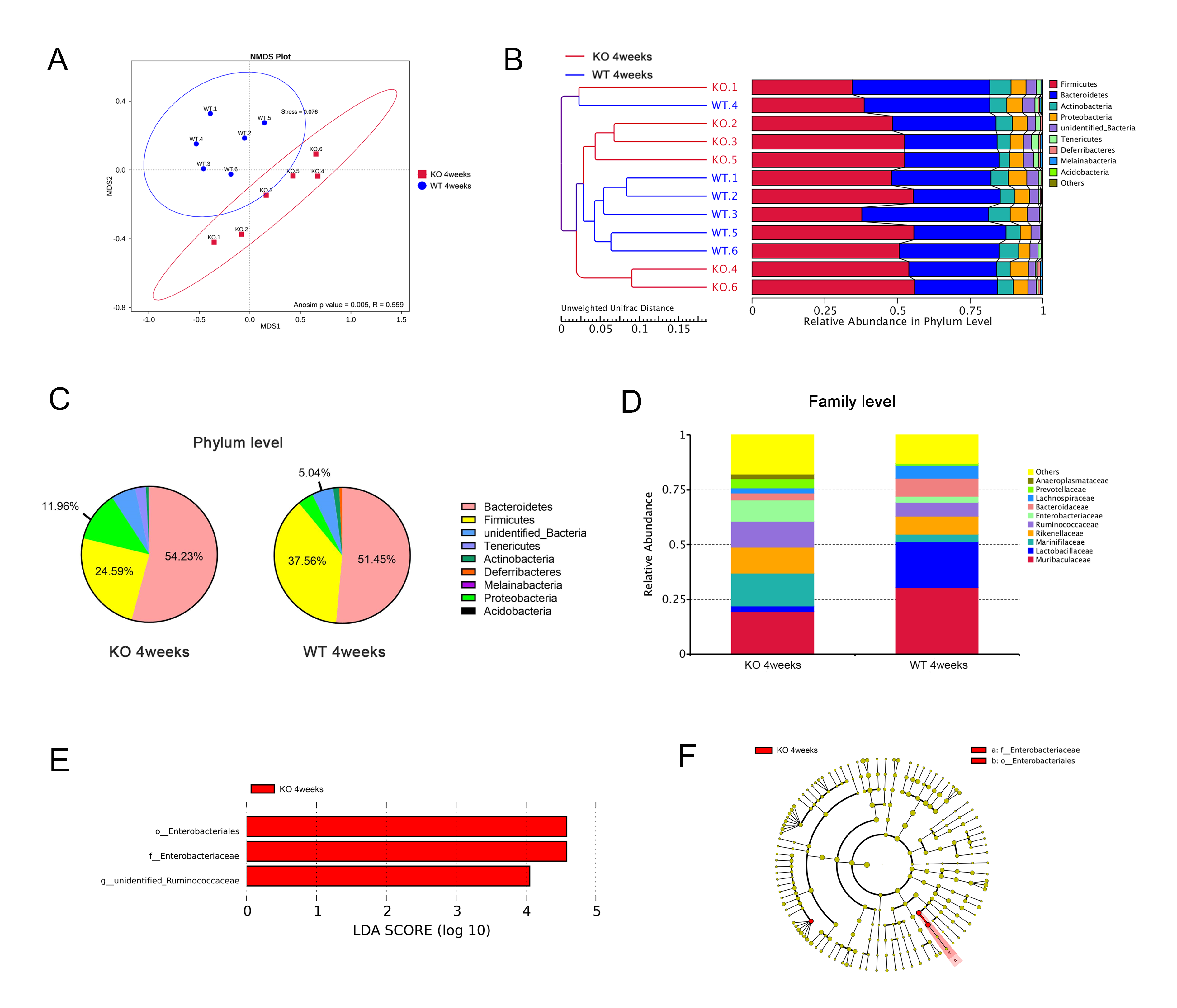

Supplement: Supplementary Figure 3 — (A) Non-metric multi-dimensional scaling (NMDS) presenting the relationship between the microbial profiles of the two groups at 4 weeks of age. (B) Unweighted pair-group method with arithmetic mean (UPGMA) based on the Unweighted UniFrac distance was used to determine the degree of similarity between the gut microbiota of WT and KO mice at 4 weeks of age (C). Fan diagram of the relative abundance of bacterial 16S rRNA genes classified at the phylum level (D). Bacterial compositions of different communities at the family level. Taxa with abundances <1% are included in Others. A significant difference (P < 0.05) was determined by one-way ANOVA followed by Tukey’s multiple comparisons test for (A). For A through (G), n = 6/group. (E,F) Linear discriminant analysis effect size (LEfSe) comparison of microbiota from faecal samples of mice at 4 weeks. LDA scores and cladogram generated from the LEfSe analysis showing the bacterial taxa that were differentially abundant in LanCL1 KO mice at 4 weeks of age. [file Image_3.TIF]

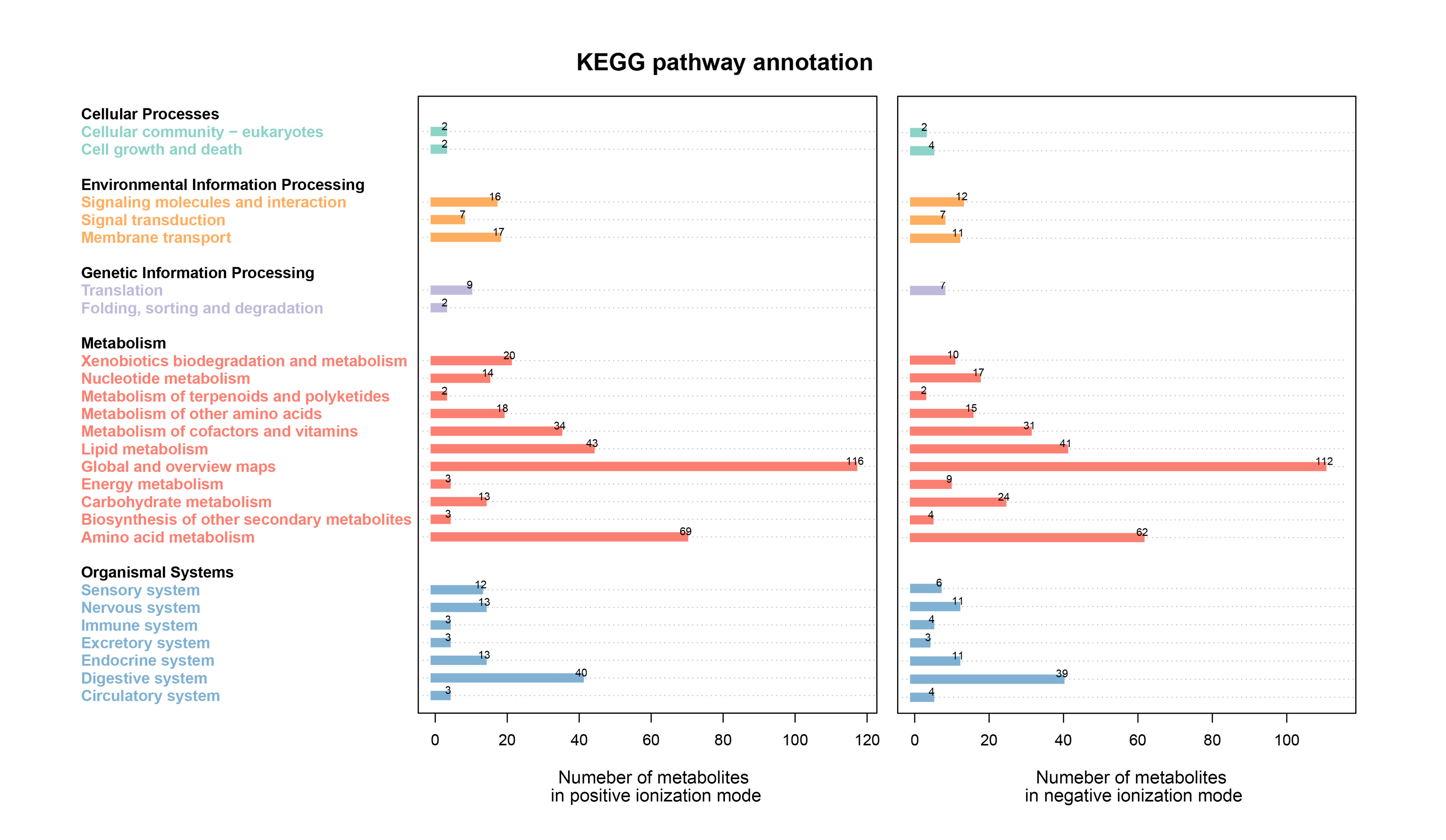

Supplement: Supplementary Figure 4 — Altered pathway annotations induced by differential metabolites in the positive and negative ionization modes. [file Image_4.TIF]
