# Supplementary material for: The Endogenous Alterations of the Gut Microbiota and Feces Metabolites Alleviate Oxidative Damage in the Brain of LanCL1 Knockout Mice
Source: Front Microbiol. 2020 Oct 7;11:557342. doi: 10.3389/fmicb.2020.557342 (PMC7575697; doi:10.3389/fmicb.2020.557342)
Supplement: Supplementary Table 1 — Gene primers used in the study. [file Table_1.DOCX]

**Supplemental Table 1. Gene primers used in the study**

| Gene | Primers |
| --- | --- |
| PGC-1α | Forward:5’-GGCCGCGTTCTTCCATTTGTGTACT-3’  Reverse:5′-TGGAGATGTCAGCCTCCTCAAAACT-3′ |
| PGC-1β | Forward:5’-GGCAGGTTCAACCCCGA-3’  Reverse:5’-CTTGCTAACATCACAGAGGATATCTTG-3’ |
| SOD-1 | Forward:5’-CAAGCGGTGAACCAGTTGTG- 3’  Reverse:5’- TGAGGTCCTGCACTGGTAC-3’ |
| SOD-2 | Forward:5’-GCCTGCACTGAAGTTCAATG-3’  Reverse:5’-ATCTGTAAGCGACCTTGCTC-3’ |
| GSTP1 | Forward:5’- ATGCCACCATACACCATTGTC-3’  Reverse:5’- GGGAGCTGCCCATACAGAC-3’ |
| GSTM1 | Forward:5’-ATACTGGGATACTGGAACGTCC-3’  Reverse:5’-AGTCAGGGTTGTAACAGAGCAT-3’ |
| GSTA4 | Forward:5’-TGATTGCCGTGGCTCCATTTA-3’  Reverse:5’-CAACGAGAAAAGCCTCTCCGT-3’ |
| Occludin | Forward: 5’-TGTGGATAAGGAACACATTTATGA-3’  Reverse: 5’-CAGACACATTTTTAACCCACTCTTCA-3’ |
| ZO-1 | Forward: 5’-TGAACGCTCTCATAAGCTTCGTAA-3’  Reverse: 5’-ACCGTACCAACCATCATTCATTG-3’ |
| GAPDH | Forward: 5’-GTTCCTACCCCCAATGTGTC-3’  Reverse: 5’- AAGGTGGAAGAGTGGGAGTT-3’ |
