# Supplementary material for: The Endogenous Alterations of the Gut Microbiota and Feces Metabolites Alleviate Oxidative Damage in the Brain of LanCL1 Knockout Mice
Source: Front Microbiol. 2020 Oct 7;11:557342. doi: 10.3389/fmicb.2020.557342 (PMC7575697; doi:10.3389/fmicb.2020.557342)
Supplement: Supplementary Table 2 — The differential feces metabolites of KO mice in positive ion mode. [file Table_2.DOCX]

**Supplemental Table 2. The** **differential feces metabolites of KO mice in positive ion mode.**

| **Name** | **FC** | **log^2^FC** | ***p* value** | **VIP** | **Up.Down** |
| --- | --- | --- | --- | --- | --- |
| **N-Acetylsphingosine** | 0.00433 | -7.85141 | 0.00000335 | 8.481288 | down |
| **(2S,3R,3aR,4aS,4bS,6aS,12bS,12cR,14aS)**  **-2-(2-Hydroxy-2-propanyl)-12b,12c-dimethyl-8,9-bis(3-methyl-2-buten-1-yl)**  **-3,3a,5,6,6a,7,12,12b,12c,13,14,14a-dodecahydro-2H,4bH-oxireno[4',4a']chromeno[5',6':6,7]**  **indeno[1,2-b]indole-3,4b-diol** | 0.006129 | -7.3502 | 0.00625 | 7.120999 | down |
| **4-(tert-Butyl)-N-{4-[3,5-bis(tert-butyl)**  **-1H-pyrazol-1-yl]phenyl}benzamideC** | 30.45483 | 4.928599 | 0.000345 | 5.550902 | up |
| **beta-D-gentiobiosyl crocetin** | 0.040045 | -4.64222 | 0.000951 | 4.874509 | down |
| **Taurine** | 0.041134 | -4.60354 | 0.003027 | 4.71629 | down |
| **Arachidonoyl amide** | 0.047284 | -4.40252 | 0.000394 | 4.625495 | down |
| **Hydrolyzed fumonisin B1** | 0.048445 | -4.36752 | 0.002344 | 4.527557 | down |
| **N-Acetylleucylleucine** | 0.058957 | -4.08419 | 0.00000589 | 4.40947 | down |
| **Tryptamine** | 0.061932 | -4.01317 | 0.000255 | 4.266224 | down |
| **3-(1H-Indol-3-yl)-N-methylpropanamide** | 0.04801 | -4.38053 | 0.008638 | 4.239302 | down |
| **N-palmitoylserinol** | 0.06703 | -3.89905 | 0.0000617 | 4.216604 | down |
| **Tyramine** | 0.062593 | -3.99785 | 0.00317 | 4.140561 | down |

*** FC = Fold change, VIP = Variable Importance in the Projection.**

*** Threshold: |log^2^FC|＞3, *p* value＜0.01 and VIP＞4.**
