# Supplementary material for: The Endogenous Alterations of the Gut Microbiota and Feces Metabolites Alleviate Oxidative Damage in the Brain of LanCL1 Knockout Mice
Source: Front Microbiol. 2020 Oct 7;11:557342. doi: 10.3389/fmicb.2020.557342 (PMC7575697; doi:10.3389/fmicb.2020.557342)
Supplement: Supplementary Table 3 — The differential feces metabolites of KO mice in negative ion mode. [file Table_3.DOCX]

**Supplemental Table 3.** **The** **differential feces metabolites of KO mice in negative ion mode.**

| **Name** | **FC** | **log2FC** | ***p* value** | **VIP** | **Up.Down** |
| --- | --- | --- | --- | --- | --- |
| **5-[(14E)-14-Nonadecen-1-yl]-1,3-benzenediol** | 263.0823 | 8.039371 | 0.000119 | 7.077322 | up |
| **Stercobilin** | 0.004456 | -7.80996 | 0.0000358 | 6.941329 | down |
| **minaprine** | 0.006261 | -7.31931 | 0.000492 | 6.381072 | down |
| **3-Sulfamoylalanine** | 0.013854 | -6.17357 | 0.00000612 | 5.521332 | down |
| **cholesteryl sulfate** | 50.59875 | 5.66103 | 0.0000523 | 5.018096 | up |
| **11(Z),14(Z)-Eicosadienoic acid** | 43.74758 | 5.451131 | 0.000567 | 4.910845 | up |
| **tolonidine** | 0.018923 | -5.72372 | 0.001743 | 4.893606 | down |
| **3-(1H-Indol-3-yl)-N-methylpropanamide** | 0.017175 | -5.86359 | 0.00414 | 4.795312 | down |
| **Caffeic acid phenethyl ester** | 0.032297 | -4.95248 | 0.000296 | 4.57695 | down |
| **Docosatrienoic acid** | 36.63839 | 5.195284 | 0.000895 | 4.574161 | up |
| **5-(Heptadec-12-enyl)resorcinol** | 24.62235 | 4.621897 | 0.002893 | 4.560786 | up |
| **4'-Methoxychalcone** | 0.033031 | -4.92002 | 0.000215 | 4.533118 | down |
| **Cedefingol** | 0.028633 | -5.12616 | 0.001018 | 4.422565 | down |
| **2-Octadecoxyethanol** | 0.034018 | -4.87758 | 0.00000491 | 4.405217 | down |
| **7-Chloro-3,5,6,8-tetramethoxy-2-(4-methoxyphenyl)-4H-chromen-4-one** | 0.035656 | -4.8097 | 0.00000128 | 4.33291 | down |
| **Taurine** | 0.032946 | -4.92377 | 0.000476 | 4.307655 | down |
| **5beta-cholanoic acid** | 27.75898 | 4.794883 | 0.003403 | 4.164436 | up |
| **ochratoxin C** | 0.044436 | -4.49213 | 0.000294 | 4.142754 | down |
| **MFCD00083370** | 22.84456 | 4.513779 | 0.000118 | 4.123606 | up |
| **(2R,3R,3aR,4aS,4bS,6aS,8aR,11aR,15bS,15cR,17aS)-3,4b-Dihydroxy-2-isopropenyl-9,9,11,11,15b,15c-hexamethyl-cc3,3a,5,6,6a,8,8a,9,11,11a,15,15b,15c,16,17,17a-hexadecahydro-2H,4bH-[2]benzofuro[5,6-e]oxireno[4',4a']chromeno[5',6':6,7]indeno[1,2-b]indol-12(7H)-one** | 0.034353 | -4.86344 | 0.005781 | 4.086196 | down |
| **Acamprosate** | 0.038559 | -4.69678 | 0.001942 | 4.061302 | down |
| **(25R)-3-Oxocholest-4-en-26-al** | 23.21734 | 4.537131 | 0.000403 | 4.008949 | up |

*** FC = Fold change, VIP = Variable Importance in the Projection.**

*** Threshold: |log^2^FC|＞3, *p* value＜0.01 and VIP＞4.**
