# Supplementary material for: The Endogenous Alterations of the Gut Microbiota and Feces Metabolites Alleviate Oxidative Damage in the Brain of LanCL1 Knockout Mice
Source: Front Microbiol. 2020 Oct 7;11:557342. doi: 10.3389/fmicb.2020.557342 (PMC7575697; doi:10.3389/fmicb.2020.557342)
Supplement: Supplementary Table 4 — The short chain fatty acids detected in feces metabolites of mice. [file Table_4.DOCX]

**Supplemental Table 4. The short chain fatty acids detected in feces metabolites of mice.**

| **Name** | **KO1** | **KO2** | **KO3** | **KO4** | **WT1** | **WT2** | **WT3** | **WT4** | **FC** | **VIP** | ***p* value** |
| --- | --- | --- | --- | --- | --- | --- | --- | --- | --- | --- | --- |
| **Propionic acid** | 22665126 | 14155309 | 18325257 | 17426280 | 12129409 | 12126404 | 10947332 | 6456425 | 1.742 | 0.743457 | 0.023565 |
| **Butyric acid** | 391120729.5 | 461865030 | 255587747.5 | 407999568.6 | 465172966 | 301204298.8 | 466186480.9 | 259628807.4 | 1.016 | 0.079318 | 0.898811 |
| **Valeric acid** | 27099723 | 30748038 | 59217118 | 65632833 | 36334334 | 42623548 | 63395538 | 13560582 | 1.172 | 0.294029 | 0.59875 |
| **Hexanoic acid** | 1719807 | 2372650 | 2011678 | 3494793 | 2890672 | 1543958 | 3034252 | 949541.4 | 1.140 | 0.277847 | 0.55526 |

*** KO = knockout, WT = wildtype, FC = Fold change, VIP = Variable Importance in the Projection.**
